# Supplementary material for: Adipose Tissue-Derived CCL5 Enhances Local Pro-Inflammatory Monocytic MDSCs Accumulation and Inflammation via CCR5 Receptor in High-Fat Diet-Fed Mice
Source: Int J Mol Sci. 2022 Nov 17;23(22):14226. doi: 10.3390/ijms232214226 (PMC9692513; doi:10.3390/ijms232214226)
Supplement: Supplementary file 1 [file ijms-23-14226-s001.zip › ijms-1983505-supplementary.pdf]

Supplementary Table S1.

Antibodies used in FACS analysis:

Antibody		
BV510- conjugated CD45	Catalog No:563891	BD Bioscience
BB515-conjugated CD11b	Catalog No:564454	BD Bioscience
BV421-conjugated F4/80	Catalog No:565411	BD Bioscience
BV421-conjugated F4/80	Catalog No:563891	BD Bioscience
Allophycocyanin(APC)-conjugated CD301	Catalog No:145708	Biolegend
PE-conjugated CD11c	Catalog No:557401	BD Bioscience
PECy7-conjugated Ly6C	Catalog No:560593	BD Bioscience
APC-conjugated Ly6G	Catalog No:560599	BD Bioscience

Supplementary Table S2.

Primer sequence used for real-time PCR:

Gene	5' Primer	3' Primer
MCP-1	GTCCCTGTCATGCTTCTGG	GCGTTAACTGCATCTGGCT
TNF-α	TACTGAACTTCGGGGTGATTGGTCC	CAGCCTTGTCCCTTGAAGAGAACC
iNOS	AATCTTGGAGCGAGTTGTGG	CAGGAAGTAGGTGAGGGCTTG
CD206	CAGGTGTGGGCTCAGGTAGT	TGTGGTGAGCTGAAAGGTGA
Arg1	CTCCAAGCCAAAGTCCTTAGAG	AGGAGCTGTCATTAGGGACATC
IL-4R	GGTCTCAACCCCCAGCTAGT	GCCGATGATCTCTCTCAAGTGAT
IL-6	TAGTCCTTCCTACCCCAATTTCC	TTGGTCCTTAGCCACTCCTTC
IL-10	GCTCTTACTGACTGGCATGAG	CGCAGCTCTAGGAGCATGTG
IL-13	CCTGGCTCTTGCTTGCCTT	GGTCTTGTGTGATGTTGCTCA
18S rRNA	ACGATGCCGACTGGCGATGC	TCCTGGTGGTGCCCTTCCGT

Supplementary Figure S1.

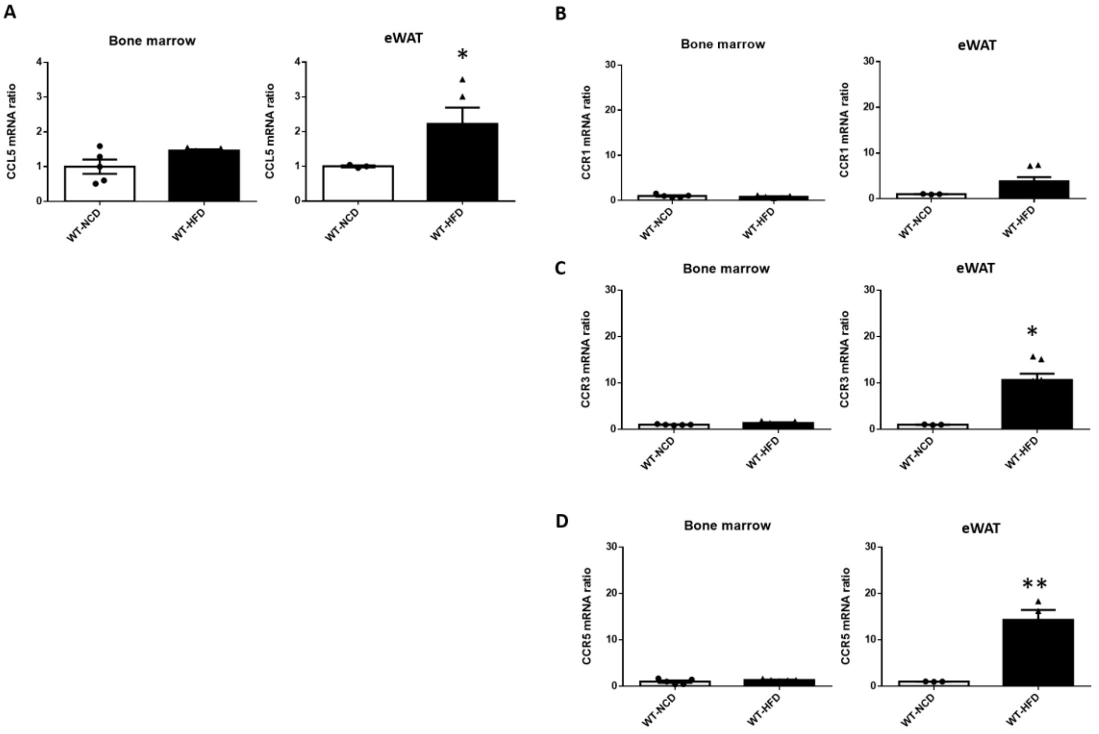

Relative mRNA level of CCL5 and its receptors CCR1, CCR3 and CCR5 in bone marrow and eWAT from WT mice fed with a NCD or HFD for 18 weeks detected by RT-qPCR. The results were normalized with S18 mRNA levels. The data are presented as mean values ± SEM from at least six mice in each group. Statistical difference is indicated: *, P < 0.05 vs. WT-NCD; **, P < 0.001 vs. WT-NCD

Supplementary Figure S2.

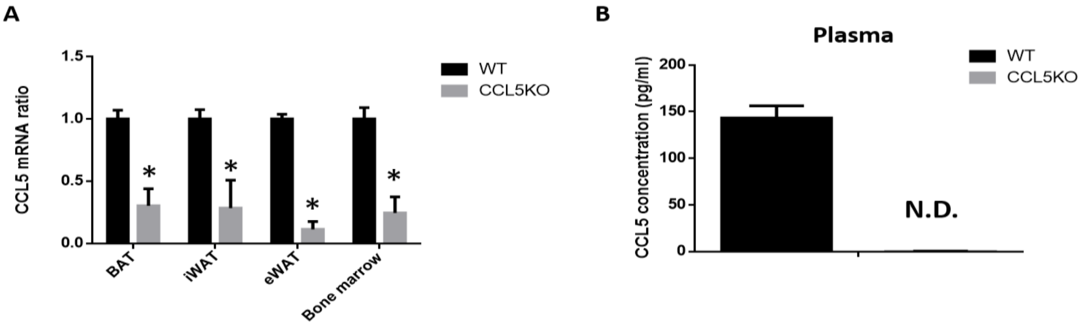

(A) Real-time PCR analysis of mRNA levels for CCL5 in BAT, iWAT, eWAT, and bone marrow of WT and CCL5KO mice. (B) Plasma concentration of CCL5 in WT and CCL5KO mice. The data are presented as mean values ± SEM from at least six mice in each group. All data presented are mean values ± SEM. *, P < 0.05 vs. WT

Supplementary Figure S3.

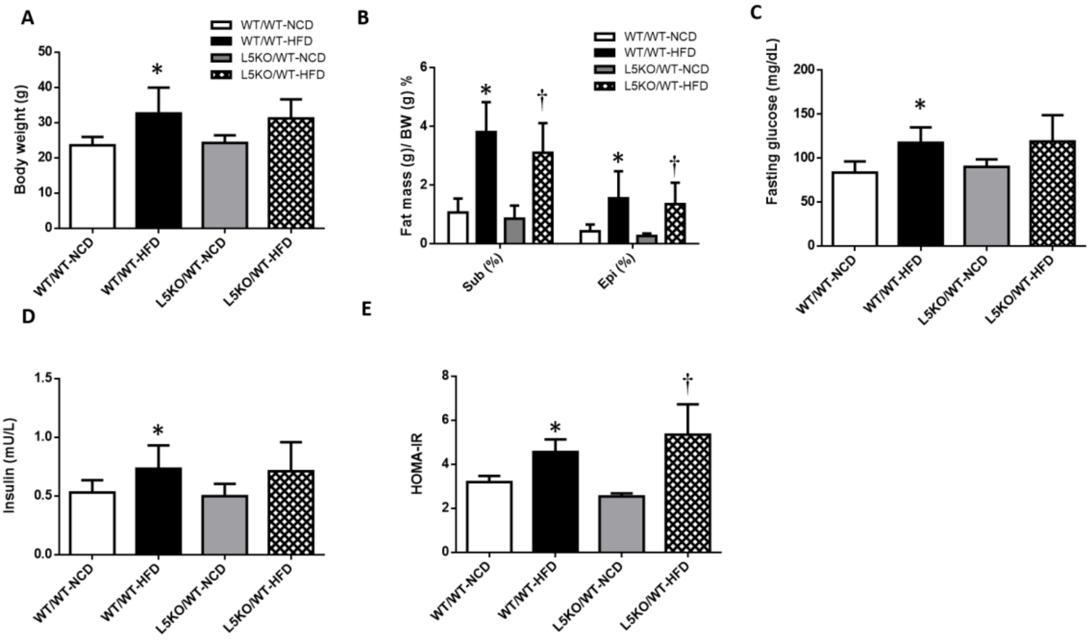

WT/WT and L5KO/WT (bone marrow-specific CCL5 knockout) mice were fed HFD (n=6-8 for each experimental group) for 20 weeks. (A) Body weights of WT/WT and L5KO/WT mice on high fat diet. (B) Subcutaneous and epidydimal fat mass per gram body weight. (C) fasting blood glucose (D) insulin (E) HOMA-IR in WT/WT and L5KO/WT mice fed with NCD and HFD diet for 20 weeks. All data are presented as mean values ± SEM from at least 6 mice in each group. Statistical difference is indicated: *, $P < 0.05$ vs. WT/WT-NCD; †, $P < 0.05$ vs. L5KO/WT-NCD.

Supplementary Figure S4.

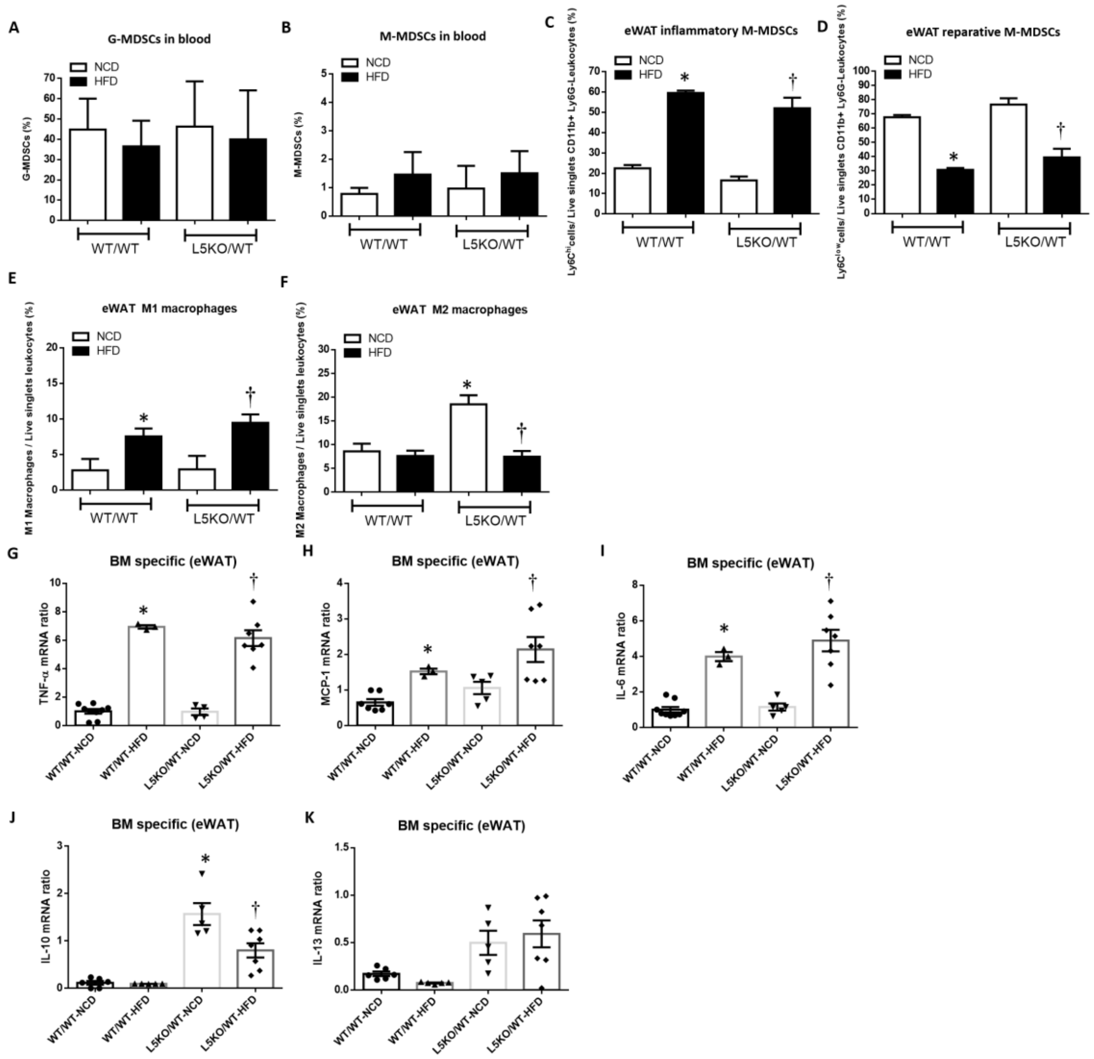

Circulating (A) G-MDSCs and (B) M-MDSCs in WT/WT and L5KO/WT mice fed with NCD and HFD diet for 20 weeks. Isolated SVF cells from eWAT were stained for CD11b, Ly6C, and Ly6G and analyzed by flow cytometry. (C) $CD11b^{+}Ly6G^{-}Ly6C^{hi}$ population represents pro-inflammatory M-MDSCs and (D) $CD11b^{+}Ly6G^{-}Ly6C^{low}$ subset represents reparative M-MDSCs (E) Analysis of $F4/80^{+}CD11c^{+}CD206^{–}$ M1 macrophages (F) $F4/80^{+}CD11c^{-}CD206^{+}$ M2 macrophages in the SVCs of the eWAT via flow cytometry. Adipose tissue protein content of (G) MCP-1, (H) TNF-α, (I) IL-6, (J) IL-10, and (K) IL-13 in WT/WT and L5KO/WT mice fed with NCD and HFD diet for 20 weeks. All data are presented as mean values ± SEM from at least 6 mice in each group. Statistical difference is indicated: *, $P < 0.05$ vs. WT/WT-NCD; †, $P < 0.05$ vs. L5KO/WT-NCD.

Supplementary Figure S5.

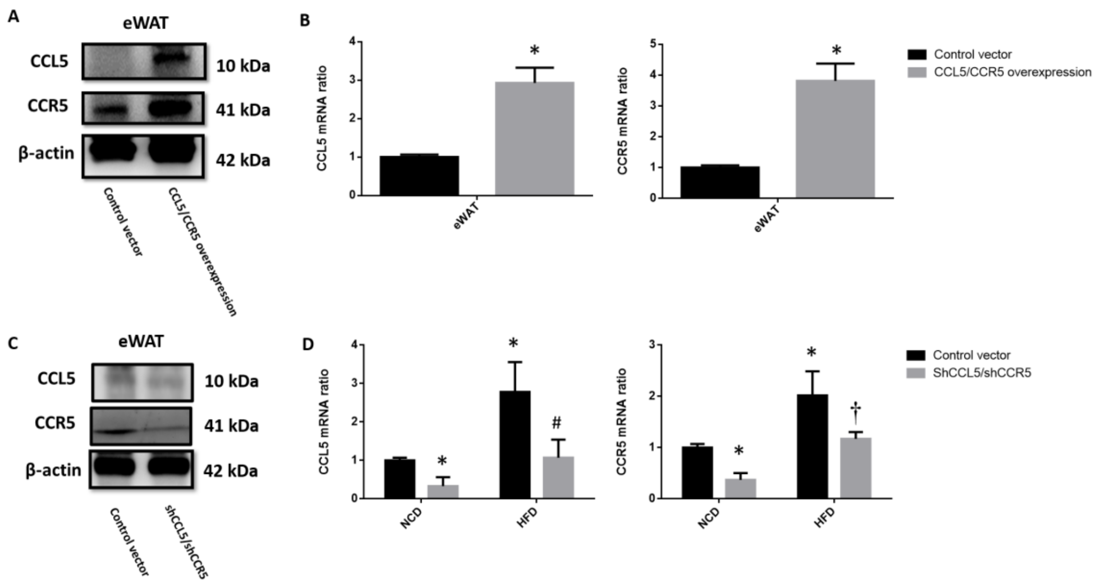

(A) Western blot analysis of eWAT CCL5 and CCR5 protein level and (B) Real-time PCR analysis of mRNA levels for CCL5 and CCR5 in eWAT of WT mice injected with scrambled or lentivirus-derived shCCL5 and shCCR5 in eWAT. (C) Western blot analysis of eWAT CCL5 and CCR5 protein level in NCD-fed WT mice injected with scrambled or lentivirus-derived shCCL5 and shCCR5 in eWAT. (D) Real-time PCR analysis of mRNA levels for CCL5 and CCR5 in eWAT in NCD and HFD-fed WT mice injected with scrambled or lentivirus-derived shCCL5 and shCCR5 in eWAT. All data presented are mean values ± SEM. *, $P < 0.05$ vs. control vector. ; #, $P < 0.05$ vs. control vector-HFD ; †, $P < 0.05$ vs. shCCL5/CCR5-NCD.
